# Adverse effects of inbreeding on the transgenerational expression of herbivore-induced defense traits in *Solanum carolinense*

**DOI:** 10.1371/journal.pone.0274920

**Published:** 2022-10-25

**Authors:** Chad T. Nihranz, Anjel M. Helms, John F. Tooker, Mark C. Mescher, Consuelo M. De Moraes, Andrew G. Stephenson

**Affiliations:** 1 Department of Biology, The Pennsylvania State University, University Park, Pennsylvania, United States of America; 2 School of Integrative Plant Sciences, Cornell University, Ithaca, New York, United States of America; 3 Department of Entomology, Texas A&M University, College Station, Texas, United States of America; 4 Department of Entomology, The Pennsylvania State University, University Park, PA, United States of America; 5 Department of Environmental Systems Science, Swiss Federal Institute of Technology, Zurich, Switzerland; University of Vienna, AUSTRIA

## Abstract

In addition to directly inducing physical and chemical defenses, herbivory experienced by plants in one generation can influence the expression of defensive traits in offspring. Plant defense phenotypes can be compromised by inbreeding, and there is some evidence that such adverse effects can extend to the transgenerational expression of induced resistance. We explored how the inbreeding status of maternal *Solanum carolinense* plants influenced the transgenerational effects of herbivory on the defensive traits and herbivore resistance of offspring. *Manduca sexta* caterpillars were used to damage inbred and outbred *S*. *carolinense* maternal plants and cross pollinations were performed to produced seeds from herbivore-damaged and undamaged, inbred and outbred maternal plants. Seeds were grown in the greenhouse to assess offspring defense-related traits (i.e., leaf trichomes, internode spines, volatile organic compounds) and resistance to herbivores. We found that feeding by *M*. *sexta* caterpillars on maternal plants had a positive influence on trichome and spine production in offspring and that caterpillar development on offspring of herbivore-damaged maternal plants was delayed relative to that on offspring of undamaged plants. Offspring of inbred maternal plants had reduced spine production, compared to those of outbred maternal plants, and caterpillars performed better on the offspring of inbred plants. Both herbivory and inbreeding in the maternal generation altered volatile emissions of offspring. In general, maternal plant inbreeding dampened transgenerational effects of herbivory on offspring defensive traits and herbivore resistance. Taken together, this study demonstrates that inducible defenses in *S*. *carolinense* can persist across generations and that inbreeding compromises transgenerational resistance in *S*. *carolinense*.

## Introduction

The environment experienced by maternal plants, including exposure to biotic and abiotic stressors, can influence the quantity and quality of resources they provide to developing seeds [1], as well as the translocation of plant signaling molecules (e.g., hormones and small RNAs) that influence offspring development [2, 3]. In turn, these can affect seed size and germination, seedling vigor, and other phenotypic traits of offspring, with often long-lasting effects on growth and reproduction [1]. In the case of herbivory, damage inflicted by herbivores and costs associated with the production of induced defenses can limit resources available for investment in seeds; however, there is also evidence that offspring of maternal plants exposed to herbivory may exhibit enhanced resistance to herbivory and stronger expression of physical and chemical defense traits [4]. Such transgenerational expression of induced resistance is plausibly adaptive, to the extent that herbivory in the maternal generation is predictive of elevated risk that offspring will also be attacked [2, 5, 6]. There is also considerable evidence that inbreeding compromises plant defense phenotypes [7–10], yet relatively few studies have examined effects of inbreeding on defensive traits in offspring [11]. The current study explores how the inbreeding status of maternal plants affects the transgenerational inheritance of induced defensive traits and herbivore resistance in outbred offspring in order to determine if inbreeding depression for anti-herbivore defensives extends across generations.

Insect herbivores are nearly omni-present in terrestrial plant populations, which can impose significant costs on plant fitness [12]. Plants have evolved a diverse array of defense strategies to mitigate the negative effects of herbivory. These include physical defenses, such as spines and trichomes [13–15], and chemical defenses, including toxins and other compounds that reduce palatability and deter feeding [15–17]. While many defenses are constitutively expressed, plants also respond to herbivory by inducing a suite of physical and chemical defenses, regulated through defensive signaling molecules and expression of defense-related genes [18, 19]. Herbivore damage can also induce the emission of volatile organic compounds (VOCs) that can repel foraging herbivores and attract natural enemies of feeding herbivores [20]. The inducibility of plant defenses allows resources to be directed primarily towards growth and reproduction when herbivore pressure is low while allowing additional investment in defenses during herbivore attack [21, 22].

Recent evidence suggests that induced defensive traits can persist across generations, with parental exposure to herbivory resulting in stronger expression of defensive traits in offspring [4, 23–28]. Previous studies have shown that herbivory by insects in one generation increases constitutive and induced expression of offspring physical defenses, such as trichomes [23, 25, 27–29], and chemical defenses, such as toxic secondary metabolites [28, 29] and volatile organic compounds [26], which, in turn, reduces herbivore performance on offspring of herbivore-damaged parent plants [24, 30]. While the mechanisms of transgenerational effects of herbivory are not fully understood, they may be caused by maternally derived epigenetic modifications of the offspring genome and/or movement of herbivore-induced hormones or small molecular RNAs from maternal tissue to developing seeds [31, 32]. Notably, transgenerational induced anti-herbivore defenses could benefit plant offspring in environments with high herbivore pressure but may adversely affect reproductive output in environments with low herbivore pressure [11].

Inbreeding is widespread among flowering plants [33] and could have adverse effects on transgenerational induction of defensive traits either by compromising defense induction in maternal plants or inhibiting signaling mechanisms that mediate the stronger expression of defense traits in offspring [9, 34]. By increasing homozygosity, inbreeding exposes deleterious recessives while also decreasing overdominance [35]. Consequently, inbreeding is frequently associated with reduced fitness of inbred relative to outbred progeny (inbreeding depression) [35]. In plants, inbreeding can reduce fitness via adverse effects on a wide range of traits related to growth and reproduction [36]. There is also considerable evidence that inbreeding can compromise plant defenses and increase susceptibility to herbivores [7, 37]. Indeed, relative to outbred plants, inbred plants frequently exhibit reduced levels of constitutive defenses against herbivores [10, 38–41], reduced ability to induce defenses in response to attack [10, 34] and reduced levels of overall resistance to herbivory [7, 42–44]. Inbreeding has also been shown to adversely affect maternal provisioning to developing seeds [45]. Yet, it is currently not known whether plant inbreeding limits the ability of maternal parents to impart inducible defenses across generations.

Previous work in our lab has shown that inbreeding in *S*. *carolinense* reduces constitutive and herbivore-induced expression of plant defenses [9, 10, 38, 46] and reduces overall resistance to insect herbivores [43, 44]. Furthermore, we previously found that herbivory of *S*. *carolinense* maternal plants has transgenerational effects on offspring growth and reproduction [47]. the current study addresses the transgenerational consequences of herbivory and maternal plant inbreeding on the defensive phenotypes of *Solanum carolinense* (L.) (Solanaceae) offspring. Specifically, we hypothesize that herbivory on *S*. *carolinense* by one of its natural herbivores, *Manduca sexta* (L.) (Lepidoptera: Sphingidae), improves the defensive phenotypes (i.e., leaf trichomes, internode spines, and volatile organic compound production) of its offspring and, therefore, adversely affects the performance of herbivores on these offspring. We also hypothesize that maternal inbreeding compromises the transgenerational effects of maternal herbivory. To our knowledge, this is the first study to address the transgenerational consequences of both herbivory and plant inbreeding on plant defenses and herbivore performance.

## Materials and methods

### Plants and insects

*Solanum carolinense* is an herbaceous perennial weed endemic to eastern North America [48]. Plant spread and propagation is facilitated by belowground horizontal rhizomes that can extend a meter or more from the parent plant [49]. *Solanum carolinense* has a multiallelic *S-*locus controlled RNAse-mediated gametophytic self-incompatibility system [50]. Plasticity in this self-incompatibility system exists and a plant’s ability to self increases when pollination and fruit set are low [51, 52]. Furthermore, different levels of self-compatibility exist depending on which *S-*alleles a plant possesses [53]. These factors allow *S*. *carolinense* to self-fertilize, and inbreeding depression has been observed in both the field and greenhouse [44, 54].

*Solanum carolinense* rhizomes were collected from plants in a large population near State College, Pennsylvania and propagated in a greenhouse to establish maternal families. Self (i.e., inbred) and cross (i.e., outbred) seeds generated from these plants were germinated and grown in a greenhouse (for details see [53]). Three maternal families of *S*. *carolinense* with distinct *S*-alleles were selected for this study [53]. Three inbred and three outbred genets from each maternal family were selected. Two ramets were produced from each genet by taking 2.5-cm rhizome cuttings and resprouting them in a pest-free growth chamber (16/8 h light/dark, 25/22˚C, 65% RH).

The tobacco hornworm (*Manduca sexta* L.) is a lepidopteran herbivore and specialist of solanaceous plants, including *S*. *carolinense* [43, 55, 56]. *Manduca sexta* eggs (Carolina Biological, Burlington, NC, USA) were hatched and larvae were reared in the laboratory for multiple generations on artificial wheat germ-based diet (Frontier Agricultural Sciences, Newark, DE, USA). To increase genetic diversity, the laboratory colony was periodically supplemented with wild *M*. *sexta* larvae collected near Rock Springs, Pennsylvania.

To examine whether there is a transgenerational effect of herbivory on defense-related traits in *S*. *carolinense* offspring and determine whether maternal plant inbreeding alters these transgenerational effects, one inbred and one outbred ramet from each genet was randomly assigned to undamaged control or herbivore-damage treatments. All plants in the herbivore-damage treatment underwent 18 sessions of feeding damage by early 4^th^ instar *Manduca sexta* larvae as previously described [11, 47]. Two larvae (not previously used) were placed on lower leaves of each plant assigned to the herbivore-damage treatment and were allowed to feed for 4 hours. Damage sessions began prior to flowering and continued until all plants produced mature fruit.

Hand pollinations were performed on 20 flowers from each maternal plant to produce seeds from herbivore-damaged and undamaged maternal plants (for details see [47]). In brief, we cross-pollinated plants in the undamaged control group with other undamaged plants and plants in the herbivore-damage treatment group with other herbivore-damaged plants. These cross-pollinations guaranteed that all offspring grown from these seeds are outbred, have a coefficient of inbreeding equal to 0 (ƒ = 0) and, on average, have the same level of heterozygosity [57]. Therefore, any consequences of inbreeding observed in offspring are the result of the maternal (not offspring) breeding status. After 12 weeks, the mature fruits were harvested, and the seeds were removed. Seeds from each genotype were planted in flats of potting soil (Pro-Mix, Premier Horticulture, Quakertown, PA, USA) in a pest-free greenhouse (16/8 h light/dark, 25/22˚C, 65% RH). After 30 days, seedlings were transplanted into 1-L pots and given 3 g Osmocote Plus fertilizer (15-9-12 NPK, plus micronutrients, Scotts Co., Marysville, OH, USA). The resulting four treatments were herbivore-damaged and undamaged offspring plants from inbred and outbred maternal plants.

### Physical defenses: Trichomes and spines

To determine whether there is a transgenerational effect of maternal herbivory and maternal inbreeding on *S*. *carolinense* physical defenses, we assessed leaf trichomes and internode spines in offspring plants. To assess leaf trichomes, images of the adaxial surface of fully developed leaves of 8-week-old offspring from the four treatments were taken with a DinoLite digital microscope (Dunwell Tech, Inc., Torrance, CA, USA). Leaf trichomes were counted using the Preview software (Apple Inc., Cupertino, CA, USA). Internode spine density, spine length, and total internode spine mass was assessed on 12-week-old offspring plants. Spines located at the third internode below the stem apex were counted and removed. Internode spine density was calculated by dividing the number of internode spines by the length of the internode. Spines were weighed with a torsion balance to determine total internode spine mass. Photos of all removed spines were taken with a DinoLite digital microscope, and spine length was measured using the DinoCapture 2.0 software (Dunwell Tech, Inc., Torrance, CA, USA).

### Chemical defenses: Volatile organic compounds (VOCs)

Because previous studies revealed that inbreeding compromises the ability of *S*. *carolinense* to induce VOCs following *M*. *sexta* damage [38, 46], we investigated the effect of maternal herbivory and maternal inbreeding on the production of VOCs in offspring. Volatile collection experiments were conducted using plants in the four treatments described above in a pest-free greenhouse equipped with high-intensity sodium-halide lights. Foliar VOCs were collected from individual leaves of 8-week-old plants by placing each leaf in a glass collection chamber (17 x 15 x 2.5 cm). We utilized a push-pull collection system that delivered charcoal-filtered air into the leaf chamber at 1.0 L min^-1^. Air inside the chamber was then pulled out at 0.8 L/min through an adsorbent volatile trap (Super-Q, 40 mg; Alltech Associates, Deerfield, IL, USA) using vacuum. Constitutively emitted and herbivore-induced VOCs were each collected continuously for 12 hours (10:00–22:00). Constitutive VOCs were collected from undamaged plants. Afterward, two newly molted third-instar *M*. *sexta* larvae (previously starved for 6 hours) were placed in each leaf chamber and allowed to feed overnight (22:00–10:00). Larvae remained in the leaf chambers and continued feeding during the subsequent herbivore-induced VOC collections. Induced VOCs were collected continuously for 12 hours following the same methods described above.

Collected volatiles were eluted from each volatile trap into a glass autosampler vial with 150 μL of dichloromethane. Nonyl acetate and n-octane were added as internal standards at concentrations of 4 and 2 ng μL^-1^, respectively. Samples were analyzed using an Agilent 5973 mass spectrometer coupled to a 6890 gas chromatograph. For analysis, 1 μL of each sample was injected and compounds were separated on an Agilent HP-1MS capillary column (30 m × 0.25 mm i.d. × 0.25 μm film thickness), using the following temperature program: 40°C for 2 min, raised at 10°C min^-1^ to 190°C followed by 12°C min^-1^ to 280°C held for 2 min, with a constant flow of helium at 0.7 mL min^-1^. Compounds were analyzed with an electron impact single quadrupole mass spectrometer (70 eV; mass scan range, 20–450 amu). Chemical data were processed using the MassHunter software (Agilent Technologies, Santa Clara, CA, USA) and tentative identification of compounds was made using MassHunter’s qualitative analysis package and the NIST14 chemical library. Structure assignments were validated by comparison of mass spectra and GC retention times with those of authentic standards. Compound abundances were corrected for leaf dry mass to estimate volatile emissions (ng/g dry weight) over the 12 h collection periods.

### Defense signaling: Jasmonic acid concentration and associated genes

To determine the transgenerational effect of maternal herbivory and maternal inbreeding on defense-related signaling hormones and gene expression, we examined constitutive and herbivore-induced levels of jasmonic acid (JA) and three JA-associated genes, a*llene oxide synthase* (AOS), *oxophytodienoate reductase-3* (OPR3), and *lipoxygenase* (LOX) in *S*. *carolinense* offspring. JA is a key phytohormone that mediates production of physical and chemical defenses associated with plant resistance to herbivores [18]. To examine constitutive levels, two leaf tissue samples (0.1 g) were collected from undamaged 12-week-old *S*. *carolinense* offspring from all treatments, flash frozen in liquid nitrogen, and stored at -80°C. To examine induced JA concentration and defense gene expression, offspring plants were fed on by two newly molted third-instar *M*. *sexta* larvae (starved for 4 hours) for 8 hours. Afterward, leaf tissue samples were taken as described previously. To quantify JA levels, endogenous phytohormones were extracted, carboxylic acids were derivatized to methyl esters, and then isolated via vapor‐phase extraction. Coupled GC/MS using isobutane chemical ionization and selected ion monitoring was used to analyze compounds [58]. Relative amounts of constitutive and induced JA were quantified by comparison with 100 ng dihydro‐JA, added as an internal standard. Retention time and spectrum was confirmed with synthetic JA standard.

RNA for quantitative real-time polymerase chain reaction (qPCR) was isolated using the RNeasy Plus kit (Qiagen Sciences, Louisville, KY, USA). One microgram of total RNA was used as a template to synthesize cDNA with the High-Capacity cDNA Reverse Transcription kit (Applied Biosystems, Foster City, CA, USA). cDNA was diluted 1:10 with water and used as a template for qPCR. Gene specific primers used for qPCR assays were designed as described previously [59], and the tomato ubiquitin gene was used as the reference gene as it has been shown to work in *S*. *carolinense* [60]. All qPCR reactions used FastStart Universal SYBR Green Master Mix (Roach Applied Science, Indianapolis, IN, USA) and were run on a 7500 Fast Real-Time PCR System (Applied Biosystems) as previously described [61].

### Herbivore performance

To examine the effects of maternal herbivory and maternal inbreeding on herbivore performance on *S*. *carolinense* offspring, we performed no-choice petri-dish and whole-plant feeding assays. In the petri-dish assays, three leaves of comparable size were removed from each offspring plant and placed in a Petri dish (100 x 15mm). One newly molted third-instar *M*. *sexta* larva (previously starved for 4 hours) was weighed and placed in each dish with a leaf. After 24 hours, larvae were removed, starved for 4 hours, and reweighed. Leaves were scanned and digitized at the beginning and end of the assay and the ImageJ v1.46 software was used to calculate total leaf area consumed. Larval relative growth rate (RGR) was calculated (change in body mass/initial mass) at the end of the bioassay. This experiment was replicated using newly molted fourth-instar *M*. *sexta* larvae.

In the whole-plant feeding assay, three newly hatched *M*. *sexta* neonates were individually placed in clip cages on 12-week-old plants. Clip cages were removed after one week and larvae were allowed to feed *ad libitum* on plants. Larvae fed until the pre-pupa stage, at which point they were put into 50-mL Falcon tubes until pupation. Days to pupation and pupae weights were recorded. Pupae were stored until adult moths eclosed. Days to eclosion and moth weights were recorded.

### Statistical analyses

Statistics were performed in R [61]. Data transformations were performed when needed to meet the assumptions of each statistical test. Non-parametric statistical tests were performed when data transformations were unable to meet the assumptions of parametric statistical models. Linear mixed-effects model ANOVAs (‘*lmer*’) were used to examine the effects of maternal herbivory (herbivore-damaged vs. undamaged), maternal breeding (outbred vs. inbred), the breeding by damage interaction, and plant maternal family on offspring physical defenses (trichome density (N = 175), and internode spine density, spine length, and internode spine mass (N = 71)), offspring hormone and gene expression (JA concentrations (N = 36) and AOS, LOX, and OPR3 expression (N = 23)), and herbivore performance traits on offspring plants (larval mass change and RGR in the no-choice assays (N = 290–314), and pupa and adult mass in the whole-plant feeding assay (N = 79–84)). Linear mixed-effects model ANCOVAs with larval initial mass as a covariate was used to assess total leaf area consumed by *M*. *sexta* larvae feeding on offspring plants (N = 290–314). All parametric models included the main effects of maternal herbivory (fixed), maternal breeding (fixed), and maternal plant family (random) and, the breeding by damage interaction effect (fixed). Linear mixed-effects model ANOVAs and ANCOVAs were also run separately for offspring of outbred and inbred maternal plants to assess the effects of maternal herbivory within each maternal breeding type. To determine the significance of random effect of maternal plant family in each model, performances for models with and without the random family effect were compared using likelihood ratio tests. Post-hoc comparisons were performed using least square means multiple comparisons (‘*lsmeans*’) to examine differences among means for all fixed interactions terms.

To analyze constitutive and herbivore-induced volatile blends, we defined a set of focal compounds. Compounds were included in analyses if they were present in at least 50% of the samples of any treatment. The total number and abundance of constitutive and induced volatiles were analyzed using a linear mixed-effect model with maternal herbivory (fixed), maternal breeding (fixed), and the breeding by damage treatment interaction (fixed) in the models (N = 28). To compare and visualize any differences in VOC blends related to maternal herbivory and maternal breeding, we conducted permutational multivariate analysis of variance (PERMANOVA) and non-metric multidimensional scaling (NMDS) separately on constitutive and induced volatile emissions of offspring. We then performed random forest analyses on constitutive and induced volatile emissions to determine the relative importance of specific compounds in distinguishing the volatile blends [62]. The quantitative differences of all identified constitutive and herbivore-induced focal compounds were analyzed separately using two-way analysis of variance (‘*lm*’).

Log-likelihood ratio tests of independence (‘*GTest*’) were performed to assess the effects of maternal herbivory, maternal breeding, and maternal plant family on survivorship of *M*. *sexta* feeding on offspring of *S*. *carolinense* in the whole-plant feeding assay (N = 108). A Wilcoxon signed-rank test (‘*wilcox*.*test*’) was performed to test the effects of maternal herbivory and maternal breeding on average time to pupation and eclosion of *M*. *sexta* in the whole-plant feeding assay and a Kruskal-Wallis test was performed to test the effect of maternal plant family on the average time to pupation and eclosion (N = 79–84). All statistical results with *P* values less than 0.1 are reported and we refer to those less than 0.05 as statistically significant. The ‘*ggplot2*’ package in R was used to create all figures [63].

## Results

### Maternal herbivory increases and maternal inbreeding decreases offspring physical defenses

**Fig 1:**
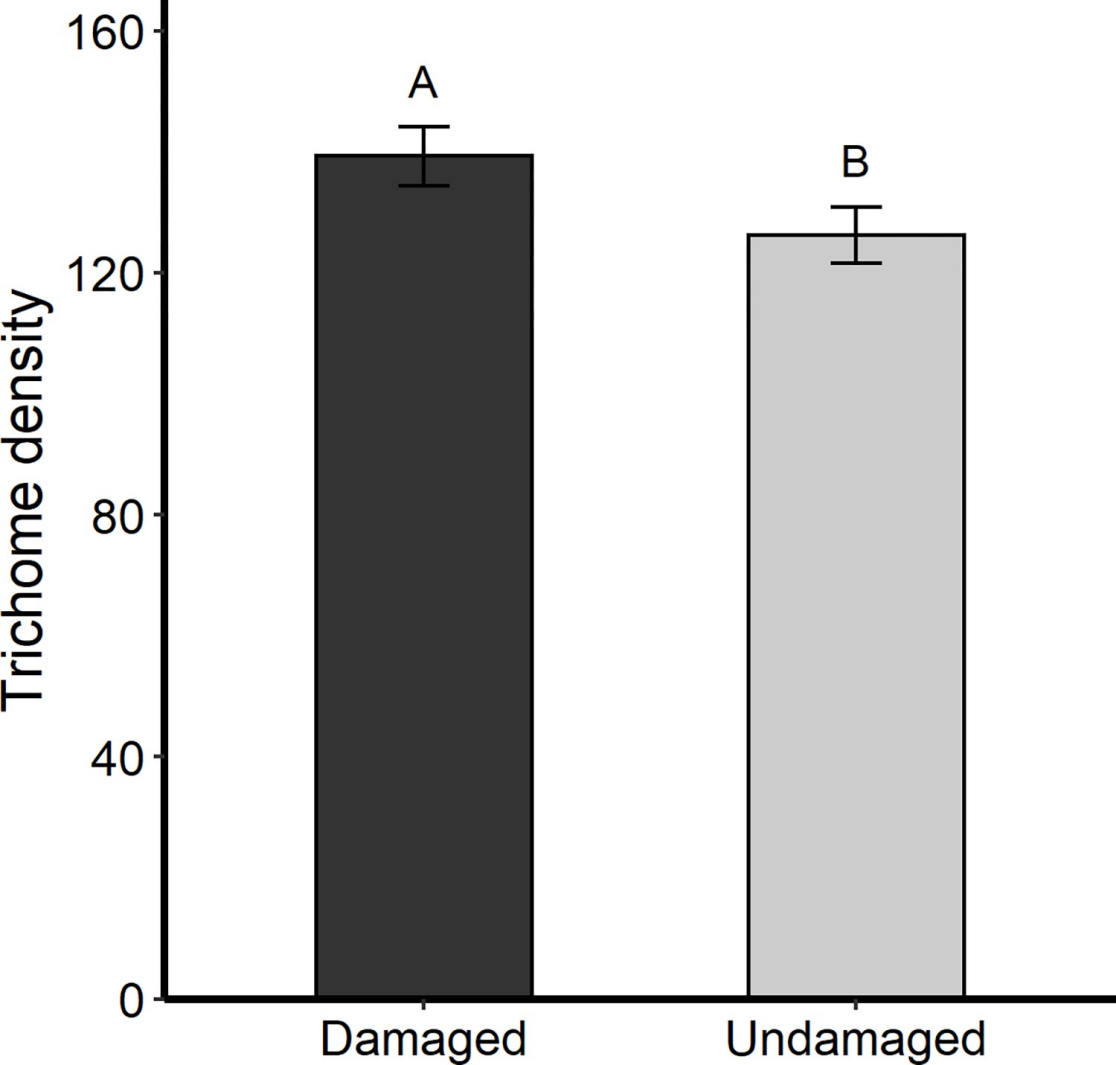
Adaxial leaf trichomes for offspring of herbivore-damaged and undamaged *S*. *carolinense* plants. Different letters indicate significant differences between maternal herbivory treatments determined by linear mixed-effects ANOVA (*P* < 0.05). Error bars correspond to standard errors.

**Fig 2:**
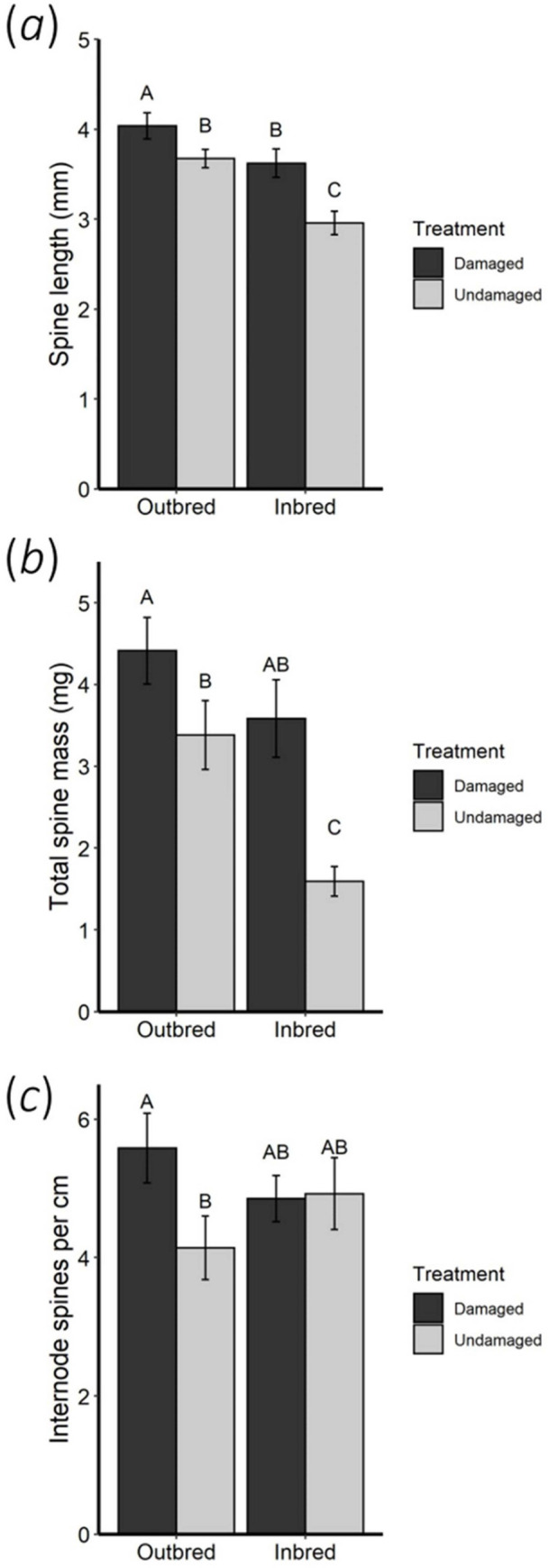
Internode spine production of *S*. *carolinense* offspring from herbivore-damaged and undamaged, inbred and outbred maternal parents. (a) Mean internode spine length, (b) total internode spine mass, and (c) internode spine density. Different letters indicate significant differences among treatments determined by post-hoc analysis using least square means multiple comparisons (*P* < 0.05). Error bars correspond to standard errors.

Offspring of herbivore-damaged plants had significantly greater adaxial leaf trichome density (Fig 1) and longer (Fig 2A) and heavier (Fig 2B) internode spines than offspring of undamaged plants (S1 Table). Offspring of herbivore-damaged plants also had greater internode spine density than offspring of undamaged plants (S1 Table; *P* = 0.066). Maternal breeding did not affect offspring leaf trichome or internode spine densities (S1 Table). However, offspring of outbred maternal plants had significantly longer and heavier internode spines than offspring of inbred maternal plants (S1 Table, Fig 2A and 2B). Maternal plant family had a significant effect on offspring leaf trichome density (χ^2^(1) = 4.702, *P* = 0.030) and internode spine density (χ^2^(1) = 10.626, *P* = 0.001), indicating that there is broad sense heritability among plants for trichome and spine production.

We observed a significant interaction of maternal herbivory and maternal breeding on offspring internode spine density (S1 Table, Fig 2C) but not on leaf trichome density, internode spine length, or spine mass (S1 Table). Comparing only offspring from outbred maternal plants, offspring of herbivore-damaged plants had significantly longer spines (*F*_1,37_ = 4.18, *P* = 0.048; Fig 2A), greater spine mass (*F*_1,37_ = 10.02, *P* = 0.051; Fig 2B), and a significantly greater internode spine density (*F*_1,37_ = 10.02, *P* = 0.003; Fig 2C) compared to offspring of undamaged outbred plants. Comparing only offspring from inbred maternal plants, offspring of herbivore-damaged plants had significantly greater leaf trichome density (*F*_1,82_ = 6.35, *P* = 0.014), spine length (*F*_1,28_ = 8.93, *P* = 0.006; Fig 2A), and spine mass (*F*_1,28_ = 11.71, *P* = 0.002; Fig 2B) compared to offspring of undamaged inbred plants.

### Maternal inbreeding reduces transgenerational effects of maternal herbivory on offspring VOC emissions

We identified 18 focal compounds from the constitutive volatile blends (S2 Table) of *S*. *carolinense* offspring and 36 compounds from the herbivore-induced volatile blends of offspring (S3 Table). NMDS analysis did not differentiate any treatment effects on the constitutive (S1 Fig; PERMANOVA, Damage: F_1,24_ = 0.708, *P* = 0.620, Breeding: F_1,24_ = 0.615, *P* = 0.680, Breeding x Damage = F_1,24_ = 0.825, *P* = 0.521) or induced (S2 Fig; PERMANOVA, Damage: F_1,24_ = 0.429, *P* = 0.831, Breeding: F_1,24_ = 0.506, *P* = 0.770, Breeding x Damage = F_1,24_ = 1.781, *P* = 0.107) volatile blends of offspring plants. Offsping herbivory significantly increased the total abundance of compounds emitted by offspring (W = 24, *P* < 0.001), as well as the number of compounds present in the induced blends (W = 4, *P* < 0.001).

#### Constitutive volatile emissions from offspring

**Fig 3:**
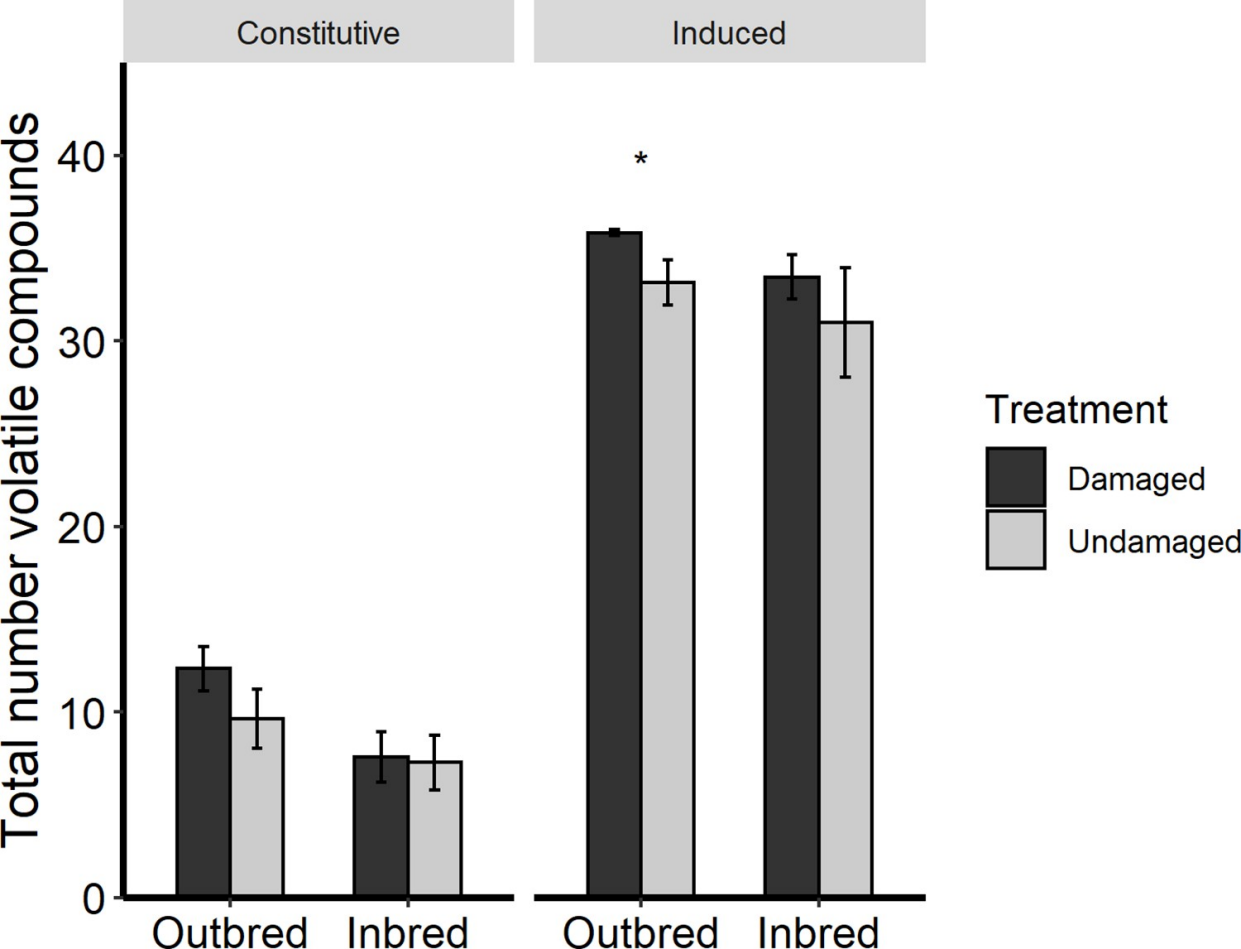
Total number of volatile organic compounds in the constitutive and induced blends of *S*. *carolinense* offspring from herbivore-damaged and undamaged, inbred and outbred maternal parents. Asterisks (*) indicate significant differences among treatments determined by Wilcox signed-rank test (*P* < 0.05). Error bars correspond to standard errors.

When comparing offspring from both breeding types, maternal herbivory had no significant effect on the total abundance of the emission (*F*_1,24_ = 0.049, *P* = 0.826; S2 Table) or the number of compounds present in the volatile blend (*F*_1,24_ = 1.034, *P* = 0.319) produced by offspring. Maternal breeding also had no effect on total abundance of constitutive emissions (*F*_1,24_ = 1.615, *P* = 0.216; S2 Table), but significantly affected the composition of the constitutive volatile blends of offspring (*F*_1,24_ = 5.343, *P* = 0.029), with offspring of outbred maternal plants producing significantly more compounds than offspring of inbred plants (Fig 3). Random forest analyses revealed (*E*)-4,8-Dimethyl-1,3,7-nonatriene (DMNT), 2-methyl-1-hepten-6-one, methyl salicylate, m-ethylacetophenone and an unknown compound (compound 25) to be the most important compounds differentiating constitutive blends based on maternal herbivory, while 2,3-heptadione and methyl salicylate were revealed to be the most important compounds differentiating constitutive blends based on maternal breeding. Offspring of herbivore-damaged plants had greater constitutive emissions of 2-methyl-1-hepten-6-one than offspring of undamaged plants (F_1,24_ = 3.029, *P* = 0.095; S2 Table). This pattern was also apparent when comparing only outbred progeny: offspring of herbivore-damaged outbred plants had significantly greater constitutive emissions of 2-methyl-1-hepten-6-one compared to offspring of undamaged outbred plants (*F*_1,12_ = 7.415, *P* = 0.019). A similar effect was not observed for offspring of inbred maternal plants (*F*_1,12_ = 0.045, *P* = 0.835). Maternal breeding also affected constitutive emissions of individual volatile compounds in *S*. *carolinense* offspring: compared to offspring of inbred plants, offspring of outbred maternal plants produced more 2-methyl-1-hepten-6-one (*F*_1,24_ = 3.246, *P* = 0.084), benzyl alcohol (W = 50, *P* = 0.016), β-ocimene (W = 65, *P* = 0.072), and decanal (*F*_1,24_ = 3.209, *P* = 0.088; S2 Table).

#### Induced volatile emissions from offspring

When comparing plants from both breeding types, maternal herbivory had no effect on the total abundance of induced emissions (*F*_1,24_ = 0.110, *P* = 0.743; S3 Table), but did affect the number of compounds present in the blend (W = 138, *P* = 0.057), with offspring of herbivore-damaged plants producing a greater number of induced compounds than offspring of undamaged plants. This pattern was also apparent when comparing only outbred progeny: offspring of herbivore-damaged outbred plants emitted significantly greater numbers of induced compounds compared to offspring of undamaged outbred plants (W = 40.5, *P* = 0.026; Fig 3); however, no similar effect was observed for offspring of inbred maternal plants (W = 29, *P* = 0.602). There was no effect of maternal breeding on the total abundance (*F*_1,24_ = 1.161, *P* = 0.292; S3 Table) or number of compounds (W = 69.5, *P* = 0.183) in the herbivore-induced volatile blends of offspring. Random forest analyses revealed decanal, epi-bicyclo-sesquiphellandrene, benzyl alcohol, phenylethyl alcohol, m-ethylacetophenone, and α-humulene as the main compounds differentiating induced blends based on maternal herbivory and unknown compound 25, α-selinene, (*Z*)-3-hexen-1-ol, phenylethyl alcohol, β-elemene, and α-guaiene as the main compounds that differentiating induced blends based on maternal breeding. Herbivore-induced emissions of β-caryophyllene and decanal were greater in offspring of herbivore-damaged maternal plants compared to offspring of undamaged maternal plants (β-caryophyllene: *F*_1,24_ = 2.933, *P* = 0.099; decanal: *F*_1,24_ = 3.296, *P* = 0.082). There was also a breeding by maternal herbivory effect on herbivore-induced emission of decanal by offspring (*F*_1,24_ = 4.003, *P* = 0.057). Comparing only offspring from outbred plants, offspring of herbivore-damaged outbred plants had significantly greater induced emissions of decanal than offspring of undamaged outbred plants (*P* = 0.013); however, no similar effect was observed for offspring of inbred maternal plants (*P* = 0.908). Maternal breeding also affected induced emissions of individual volatile compounds in *S*. *carolinense* offspring: offspring of outbred plants had greater herbivore-induced volatile emissions of β-elemene (*F*_1,24_ = 3.296, *P* = 0.073) and an unknown (compound 25) (*F*_1,24_ = 2.953, *P* = 0.099) compared to offspring of inbred plants.

#### Maternal herbivory and maternal inbreeding do not affect JA concentrations or expression of JA-associated genes

Constitutive and herbivore-induced concentrations of jasmonic acid (JA) in *S*. *carolinense* offspring were not affected by maternal herbivory, maternal breeding, or their interaction (S4 Table). There were significant differences in constitutive JA concentrations among maternal plant families (χ^2^(1) = 8.765, *P* = 0.003), but not in induced JA concentrations (χ^2^(1) = 0.001, *P* = 0.976). Constitutive and induced expression of AOS, LOX, and OPR3 gene transcripts in offspring were not affected by maternal herbivory, maternal breeding, or their interaction (S4 Table).

#### Maternal herbivory and maternal inbreeding alter *M*. *sexta* performance on offspring

In no-choice petri-dish assays with third-instar *M*. *sexta* larvae, maternal herbivory did not affect total leaf area consumed (Table 1), larval mass gain (S5 Table), or larval RGR (S5 Table). There was an effect of maternal herbivory on larval mass gain when comparing only offspring of outbred plants: larvae gained more mass feeding on leaves of offspring from undamaged outbred plants compared to offspring of herbivore-damaged outbred plants (*F*_1,154_ = 2.80, *P* = 0.096). However, no similar effect was observed for larvae feeding on leaves from offspring of undamaged or herbivore-damaged inbred plants (*F*_1,131_ = 0.237, *P* = 0.628). Maternal inbreeding increased the total leaf area consumed by third-instar larvae (Table 1) and larval mass gain (*F*_1,285_ = 3.320, *P* = 0.069; S5 Table) but did not affect larval RGR (S5 Table). In no-choice petri-dish assays with fourth-instar *M*. *sexta* larvae, larvae consumed significantly more leaf area on offspring of inbred plants compared to outbred offspring (Table 1). However, total leaf area consumed, larval mass gain, and RGR were not affected by maternal herbivory, maternal plant family (χ^2^(1) = 0, *P* = 1) or the interaction between maternal herbivory and maternal breeding (Table 1, S5 Table).

**Table 1:** Linear mixed-effects ANCOVAs for the effects of maternal herbivory (damage), maternal breeding, and their interaction on the total leaf area consumed by third and fourth instar *M*. *sexta* larvae.

*M*. *sexta instar*	*Source of variation*	*Df*	*SS*	*F*	*P*
Third instar	Larval initial mass	1	13.364	56.756	**< 0.001**
	Damage	1	0.232	0.985	0.322
	Breeding	1	0.682	2.896	0.089
	Breeding x Damage	1	0.121	0.512	0.475
	Error	285	67.106		
Fourth instar	Larval initial mass	1	39.566	69.779	**< 0.001**
	Damage	1	0.112	0.198	0.657
	Breeding	1	3.599	6.347	**0.012**
	Breeding x Damage	1	0.141	0.249	0.618
	Error	309	175.203		

*P* values <0.05 are in boldface.

In whole-plant feeding assays, *M*. *sexta* larvae that fed on *S*. *carolinense* offspring from outbred maternal plants had significantly greater pupa mass than *M*. *sexta* that fed on offspring of inbred maternal plants (Table 2; S3 Fig). Days to pupation was not affected by maternal herbivory (W = 918.5, *P* = 0.742), maternal breeding (W = 735.5, *P* = 0.188), or maternal plant family (H = 0.091, df = 2, *P* = 0.635). Survivorship to adulthood of *M*. *sexta* larvae feeding on *S*. *carolinense* offspring was also not affected by maternal herbivory, maternal breeding, breeding by damage interaction, or maternal plant family (S6 Table). *Mandcua sexta* that fed on offspring of undamaged plants eclosed significantly earlier than those that fed on offspring of herbivore-damaged plants (W = 981.5, *P* = 0.046; S4 Fig). However, days to eclosion was not affected by maternal breeding (W = 677, *P* = 0.315; S4 Fig) or maternal plant family (H = 0.045, df = 2, *P* = 0.978). Adult mass of *M*. *sexta* that fed on *S*. *carolinense* offspring was not affected by maternal herbivory (Table 2); however, maternal breeding did significantly affect adult mass with greater adult mass of *M*. *sexta* that feed on offspring of outbred plants (Table 2).

**Table 2:** Linear mixed-effects ANOVA for the effects of maternal herbivory (damage), maternal breeding, and their interaction on pupa and adult mass of *M*. *sexta* feeding on offspring of *S*. *carolinense* in the whole plant feeding assay.

*Traits*	*Source of variation*	*Df*	*SS*	*F*	*P*
Pupa mass	Damage	1	0.396	0.902	0.345
	Breeding	1	4.214	9.595	**0.003**
	Breeding x Damage	1	1.184	2.696	0.105
	Error	78	34.258		
Adult mass	Damage	1	0.171	0.881	0.351
	Breeding	1	1.291	6.643	**0.012**
	Breeding x Damage	1	0.060	0.310	0.579
	Error	75	14.573		

*P* values <0.05 are in boldface.

## Discussion

Our findings indicate maternal herbivory on *S*. *carolinense* plants influences the defense traits of offspring: compared to offspring of undamaged plants, offspring of herbivore-damaged plants had greater constitutive physical defenses (i.e., leaf trichome density, and spine density, mass and length), produced a greater number of herbivore-induced volatile compounds, and had greater herbivore resistance (i.e., reduced herbivore performance). We also observed transgenerational effects of maternal breeding: compared to the offspring of outbred plants, the offspring of inbred maternal plants exhibited reduced expression of defense traits (i.e., leaf trichomes, internode spines, and volatile organic compounds) and increased susceptibility to herbivory. We also found that maternal inbreeding dampened the transgenerational effects of herbivory on offspring defensive phenotypes and resistance to herbivores. Taken together, our data reveals significant transgenerational impacts of maternal herbivory and maternal breeding on plant physical defenses and volatile emissions, as well as on the resistance of offspring plants to herbivory.

### Maternal herbivory increases the expression of anti-herbivore defense traits in offspring

It is well known that herbivory induces physical and chemical defenses in most plant species [22, 64] and there is growing evidence that herbivory in one generation can affect defensive traits in plant offspring [4, 23–28, 65]. The current study reveals effects of herbivory in the maternal generation on both physical and chemical defensive traits of offspring. To date, studies of herbivore-mediated transgenerational effects on physical plant defenses have primarily focused on leaf trichomes. For example, mechanically wounded *Mimulus guttatus* had offspring with increased constitutive trichome density [23, 25, 27] and caterpillar herbivory on *Arabidopsis thaliana* increased leaf trichome density in offspring [66]. To our knowledge, our study is the first to demonstrate a transgenerational impact of insect herbivory on plant spines. Trichomes and spines generally deter herbivore feeding, limit herbivore movement, and limit herbivore access to feeding sites [13, 14, 67–69]. Recent evidence shows that non-glandular leaf trichomes of *S*. *carolinense* cause extensive damage to *M*. *sexta* peritrophic matrix [10, 70], a membrane lining the caterpillar gut that aids in digestion and acts as a barrier against pathogen infection [71]. Leaf and internode spines have also been shown to reduce the rate of herbivory and leaf tissue loss in a variety of plant species [14], including *S*. *carolinense* and other *Solanum* species [72].

Plants also respond to insect feeding by increasing production of volatile organic compounds (VOCs), which can be toxic or repellent to herbivores and frequently mediate attraction of predators and parasitoids of feeding herbivores [20, 73, 74]. Previous work has demonstrated that herbivory by *M*. *sexta* caterpillars on *S*. *carolinense* plants influences the quantity and composition of VOC emissions [46]. In the current study, *M*. *sexta* herbivory on maternal plants increased the number of VOCs emitted from undamaged offspring and influenced the emissions of single compounds within both the constitutive (e.g., 2-methyl-1-hepten-6-one) and herbivore-induced (e.g., β-caryophyllene) volatile blends of offspring. Although no previous studies have characterized the ecological role of 2-methyl-1-hepten-6-one in plant-insect interactions, emission of this compound was altered by maternal herbivory, suggesting it could have a defensive role. β-caryophyllene has been reported as an attractant for several herbivore natural enemies, including green lacewing [75], parasitoids [76, 77], and entomopathogenic nematodes [78]. A recent study reported that transgenerational effects of herbivory on VOC emissions in *Brassica rapa* offspring were transient in offspring and completely disappeared by the second generation after herbivory [27]. Therefore, it is possible our observed transgenerational effect of herbivory on the quantitative production of VOCs is due to the age of the plants at collection time. Additional studies are needed to determine what, if any, impact these subtle differences in VOC composition have on herbivore preference or behavior of natural enemies.

Induction of physical and chemical defenses within one generation of a plant can negatively affect herbivore performance [79, 80]. Consequently, we hypothesized that transgenerational induction of defenses would increase herbivore resistance in offspring of herbivore-damaged maternal plants. Our data show that herbivory of maternal *S*. *carolinense* plants negatively affects *M*. *sexta* larvae performance (i.e., mass gain, time to adulthood) on offspring. Mass gain of insects is often correlated with adult fitness [81] and longer developmental times can increase predation risk for insects [82]; therefore, transgenerational effects of maternal herbivory have potential to directly affect herbivore development and to increase exposure of herbivores to natural enemies. If maternal environment is an accurate predictor of offspring environment, and if maternal plants can modulate offspring defensive phenotypes, we would expect to find transgenerational effects of herbivory that improve offspring resistance and fitness [29, 31, 83, 84]. This could be particularly beneficial in environments with high herbivore pressure where increased expression of defensive traits would provide offspring with increased resistance to herbivory.

### Maternal inbreeding compromises offspring defenses

There is a growing body of evidence indicating that inbreeding negatively affects plant defensive traits and herbivore resistance, and compromises the induction of plant defenses against herbivores [7, 9, 85, 86]. In *S*. *carolinense*, inbreeding adversely affects both physical and chemical defenses [10, 38, 40, 46], resulting in greater amounts of herbivore damage under field conditions [43, 44, 54], increased oviposition by *M*. *sexta* adults [87], reduced recruitment of herbivore natural enemies upon herbivore attack [46], and lower induction of anti-herbivore defenses [10, 11, 34]. Here, we show that the adverse effects of maternal inbreeding on plant defenses extend to the outbred offspring of inbred plants. We found that maternal inbreeding in *S*. *carolinense* compromised physical defenses (i.e., spine size and mass) and rendered plants less resistant to a native herbivore. Our data also indicate that maternal inbreeding reduces volatile emissions of *S*. *carolinense* offspring, as offspring of inbred plants produced fewer constitutive compounds and reduced the abundance of individual compounds in their induced and constitutive blends compared to offspring of outbred plants. Taken together, our data shows that maternal inbreeding can compromise physical and chemical defenses even in outbred offspring.

### Maternal inbreeding dampens transgenerational effects of herbivory

Inbreeding is common in flowering plants and the adverse effects of inbreeding on seed number and offspring performance, termed inbreeding depression, are well-documented since the time of Darwin [88]. In fact, inbreeding depression is considered to be the driving force in the evolution of floral traits, mating patterns, and breeding systems in flowering plants [33, 35, 36]. Our findings suggest that maternal inbreeding reduces expression of defense traits and herbivore resistance in offspring of herbivore-damaged plants. For example, we found that maternal herbivory resulted in increased spine density and number of herbivore-induced VOCs in offspring from outbred maternal plants, but not in offspring of inbred maternal plants. Furthermore, transgenerational effects of herbivory on the number of compounds in the induced volatile blends of offspring and emissions of individual volatile compounds (e.g., 2-methyl-1-hepten-6-one) were more pronounced in offspring of outbred maternal plants, indicating that maternal plant inbreeding has potential to influence induction of plant traits involved in indirect defenses across generations. The adverse effects of inbreeding on ecologically relevant defensive traits (e.g., spines and VOCs) could compromise plant resistance to herbivory by decreasing direct defenses against feeding herbivores, as well as through alteration of indirect chemical defenses that aid in attracting parasitoids or other herbivore natural enemies to host plants. Indeed, we found that maternal herbivory negatively influenced mass gain of *M*. *sexta* caterpillars on offspring from outbred plants, but not on offspring from inbred plants. Taken together, our data show that negative effects of maternal inbreeding in *S*. *carolinense* extend across generations, compromising offspring defensive phenotypes and herbivore resistance even in the outbred offspring produced by inbred maternal plants. If the reduced resistance to herbivory impacts reproductive output of the offspring of inbred maternal parents, these findings strongly suggest that the magnitude of inbreeding depression (a key determinate in most models of the evolution on plant breeding systems) may be vastly underestimated unless the transgenerational impacts are also assessed.

### Potential mechanisms mediating transgenerational effects of herbivory and inbreeding

Jasmonic acid (JA) regulates induced plant defenses associated with plant resistance to herbivory [18]. Moreover, inbreeding has been shown to reduce the expression of genes located in the JA biosynthetic pathway of *S*. *carolinense* [8, 9] and thereby compromise host plant defenses. In addition to the role of JA in mediating induced plant defenses to herbivory, some research suggests that JA is also involved in transgenerational plant defense responses to herbivory [24]. Despite this, we did not find any differences in constitutive or induced JA concentrations or defense-related gene expression in *S*. *carolinense* offspring from herbivore-damaged and undamaged plants, suggesting mechanisms other than phytohormone signaling may underlie the transgenerational impact of *M*. *sexta* herbivory in *S*. *carolinense*.

There is increasing evidence that epigenetic modifications mediate transgenerational effects of environmental stress in plant offspring [2, 4, 24, 27, 31]. Epigenetic modifications (e.g., DNA methylation, histone modification, and small-RNA activity) change plant gene expression and can be altered by herbivory [27, 89, 90]. Furthermore, there is evidence that epigenetic modifications are associated with inbreeding depression in plants. For example, inbred plants of *Scabiosa columbaria* had significantly greater levels of DNA methylation compared to outbred plants and the adverse effects of inbreeding disappeared when inbred plants were treated with a demethylation agent, further indicating an epigenetic role in inbreeding depression in plants [91].

It seems reasonable to predict that epigenetic modifications control the transgenerational effects of *M*. *sexta* herbivory and maternal inbreeding of *S*. *carolinense* on defensive phenotypes of offspring and their resistance to herbivores. Epigenetic modifications may allow phenotypic plasticity in defense traits across generations without modifying the plant genome and modulate offspring investment in resistance according to herbivore pressure. Furthermore, these epigenetic modifications may be adaptive if they contribute to increased offspring fitness in environments with high herbivore pressure and have limited reproductive costs when herbivore pressure is low. Future work should examine the contribution of epigenetic mechanisms to transgenerational responses of plants to insect herbivory, determine the impact of inbreeding on these mechanisms, and quantify the impact on offspring reproduction.

## Supporting information

S1 Table Offspring physical defenses. Linear mixed-effects ANOVAs for the effects of maternal herbivory (damage), maternal breeding, and their interaction on trichome density, spine density, spine length, and total internode spine mass. *P* values <0.05 are in boldface.
(DOCX)

S2 Table Focal compounds from constitutive volatile emissions of *S*. *carolinense* offspring. Compounds are in alphabetical order. Differences in quantities between maternal herbivory treatments and maternal breeding were determined by two-way ANOVAs and boldface indicate *P* < 0.1.
(DOCX)

S3 Table Focal compounds from induced volatile emissions of *S*. *carolinense* offspring. Compounds are in alphabetical order. Differences in quantities between maternal herbivory treatments and maternal breeding were determined by two-way ANOVAs and boldface indicate *P* < 0.1.
(DOCX)

S4 Table Jasmonic acid and JA-associated genes. Linear mixed-effects ANOVA for the effects of maternal herbivory (damage), maternal breeding, and their interaction on constitutive and induced levels of jasmonic acid (JA) and three JA-associated genes, a*llene oxide synthase* (AOS), *oxophytodienoate reductase-3* (OPR3), and *lipoxygenase* (LOX) in *S*. *carolinense* offspring. There were no significnat differenes.
(DOCX)

S5 Table Larval mass gain and relative growth rate. Linear mixed-effects ANOVAs for the effects of maternal herbivory (damage), maternal breeding, and their interaction on larval mass gain and relative growth rate (RGR) of third and fourth instar *M*. *sexta* larvae feeding on leaves of *S*. *carolinense* offspring. There were no significant differences.
(DOCX)

S6 Table *Manduca sexta* survivorship. Log-likelihood ratio test of independence for the effects of maternal herbivory (damage), maternal breeding, their interaction, and maternal plant family on survivorship to adulthood of *M*. *sexta* feeding on *S*. *carolinense* offspring. There were no significant differences.
(DOCX)

S1 Fig Non-metric multi-dimensional scaling (NMDS) of constitutive volatile organic compounds (VOCs) emitted from offspring of herbivore-damaged and undamaged, inbred and outbred maternal *S*. *carolinense* plants. S-DAM = offspring of herbivore-damaged inbred maternal plants, S-UD = offspring of undamaged inbred maternal plants, X-DAM = offspring of herbivore-damaged outbred maternal plants, and X-UD = offspring of undamaged outbred maternal plants.
(TIF)

S2 Fig Non-metric multi-dimensional scaling (NMDS) of induced volatile organic compounds (VOCs) emitted from offspring of herbivore-damaged and undamaged, inbred and outbred maternal *S*. *carolinense* plants. S-DAM = offspring of herbivore-damaged inbred maternal plants, S-UD = offspring of undamaged inbred maternal plants, X-DAM = offspring of herbivore-damaged outbred maternal plants, and X-UD = offspring of undamaged outbred maternal plants.
(TIF)

S3 Fig Pupa and adult mass of *M*. *sexta* that fed on offspring of herbivore-damaged and undamaged, inbred and outbred maternal *S*. *carolinense* plants. Different letters indicate significant differences among breeding by damage treatments determined by post hoc analysis using least square means multiple comparisons (*P* < 0.05). Error bars correspond to standard errors.
(TIF)

S4 Fig Days to eclosion of *M*. *sexta* that fed on offspring of (A) herbivore-damaged and undamaged and (B) inbred and outbred maternal *S*. *carolinense* plants. Different letters indicate significant differences among maternal breeding by maternal herbivory treatments determined by Wilcox signed-rank tests (*P* < 0.05). Error bars correspond to standard errors.
(TIF)
