# Supplementary material for: Adverse effects of inbreeding on the transgenerational expression of herbivore-induced defense traits in Solanum carolinense
Source: PLoS One. 2022 Oct 25;17(10):e0274920. doi: 10.1371/journal.pone.0274920 (PMC9595541; doi:10.1371/journal.pone.0274920)
Supplement: S1 Table — Linear mixed-effects ANOVAs for the effects of maternal herbivory (damage), maternal breeding, and their interaction on trichome density, spine density, spine length, and total internode spine mass. P values <0.05 are in boldface. (DOCX) [file pone.0274920.s001.docx]

**S1 Table. Offspring physical defenses.** Linear mixed-effects ANOVAs for the effects of previous generation herbivore damage, maternal breeding type, and their interaction on trichome density, spine density, spine length, and total internode spine mass. *P* values <0.05 are in boldface.

| *Plant trait* | *Source of variation* | *Df* | *SS* | *F* | *P* |
| --- | --- | --- | --- | --- | --- |
| Trichome density | Damage | 1 | 0.090 | 4.406 | **0.037** |
|  | Breeding | 1 | 0.001 | 0.040 | 0.841 |
|  | Breeding x Damage | 1 | 0.047 | 2.295 | 0.132 |
|  | Error | 170 | 3.470 |  |  |
|  |  |  |  |  |  |
| Spine density | Damage | 1 | 0.087 | 3.492 | 0.066 |
|  | Breeding | 1 | 0.003 | 0.121 | 0.729 |
|  | Breeding x Damage | 1 | 0.179 | 7.177 | **0.009** |
|  | Error | 66 | 1.644 |  |  |
|  |  |  |  |  |  |
| Spine length | Damage | 1 | 4.448 | 13.088 | **< 0.001** |
|  | Breeding | 1 | 5.229 | 15.384 | **< 0.001** |
|  | Breeding x Damage | 1 | 0.462 | 1.360 | 0.248 |
|  | Error | 66 | 22.431 |  |  |
|  |  |  |  |  |  |
| Total spine mass | Damage | 1 | 3.065 | 14.107 | **< 0.001** |
|  | Breeding | 1 | 2.588 | 11.912 | **< 0.001** |
|  | Breeding x Damage | 1 | 0.285 | 1.317 | 0.255 |
|  | Error | 66 | 14.339 |  |  |
