# Supplementary material for: Adverse effects of inbreeding on the transgenerational expression of herbivore-induced defense traits in Solanum carolinense
Source: PLoS One. 2022 Oct 25;17(10):e0274920. doi: 10.1371/journal.pone.0274920 (PMC9595541; doi:10.1371/journal.pone.0274920)
Supplement: S2 Table — Compounds are in alphabetical order. Differences in quantities between maternal herbivory treatments and maternal breeding were determined by two-way ANOVAs and boldface indicate P < 0.1. (DOCX) [file pone.0274920.s002.docx]

**S2 Table.** **Focal compounds from constitutive volatile emissions of *S. carolinense* offspring.** Compounds are in alphabetical order. Differences in quantities between paternal damage treatments and maternal breed type were determined by two-way ANOVAs and boldface indicate *P* < 0.1.

|  | **Parental treatment** | | **Maternal breeding type** | |
| --- | --- | --- | --- | --- |
| **Compound** | **Damaged** | **Undamaged** | **Outbred** | **Inbred** |
| 2,3-heptanedione | 0.06 ± 0.03 | 0.05 ± 0.02 | 0.07 ± 0.02 | 0.03 ± 0.02 |
| 2-methyl-1-hepten-6-one | **0.62 ± 0.20** | **0.31 ± 0.12** | **0.62 ± 0.19** | **0.29 ± 0.12** |
| α-farnesene | 0.34 ± 0.24 | 0.34 ± 0.21 | 0.53 ± 0.30 | 0.15 ± 0.08 |
| β-ocimene | 0.67 ± 0.21 | 6.95 ± 5.76 | **7.37 ± 6.16** | **0.70 ± 0.38** |
| benzyl alcohol | 0.10 ± 0.04 | 0.20 ± 0.13 | **0.25 ± 0.14** | **0.06 ± 0.03** |
| caryophyllene oxide | 0.04 ± 0.02 | 0.05 ± 0.04 | 0.08 ± 0.04 | 0.02 ± 0.01 |
| compound 14 | 0.04 ± 0.02 | 0.08 ± 0.05 | 0.09 ± 0.05 | 0.03 ± 0.03 |
| compound 25 | 0.05 ± 0.01 | 0.13 ± 0.12 | 0.16 ± 0.12 | 0.03 ± 0.01 |
| decanal | 0.01 ± 0.01 | 0.02 ± 0.01 | **0.02 ± 0.01** | **0.01 ± 0.01** |
| (*E*)-3-hexen-1-ol | 0.64 ± 0.36 | 7.94 ± 6.38 | 8.53 ± 6.83 | 0.58 ± 0.24 |
| (*E*)-4,8-dimethylnona-1,3,7-triene | 31.28 ± 8.76 | 53.99 ± 23.01 | 61.93 ± 24.07 | 24.96 ± 8.05 |
| geraniolene | 3.99 ± 1.39 | 22.03 ± 14.53 | 25.19 ± 15.38 | 2.12 ± 0.88 |
| geranylacetone | 0.03 ± 0.02 | 0.04 ± 0.02 | 0.06 ± 0.03 | 0.01 ± 0.01 |
| methyl salicylate | 11.75 ± 5.55 | 43.68 ± 27.73 | 52.79 ± 29.26 | 4.92 ± 2.87 |
| m-ethylacetophenone | 0.09 ± 0.02 | 0.11 ± 0.02 | 0.11 ± 0.02 | 0.09 ± 0.02 |
| nerolidol | 0.33 ± 0.10 | 0.98 ± 0.56 | 1.16 ± 0.59 | 0.19 ± 0.05 |
| (*Z*)-3-hexen-1-ol | 0.05 ± 0.03 | 0.73 ± 0.67 | 0.79 ± 0.72 | 0.04 ± 0.02 |
|  |  |  |  |  |
| **Average** | 9.77 ± 1.12 | 8.53 ± 1.10 | 10.79 ± 1.08 | 7.43 ± 0.10 |
