# Supplementary material for: Adverse effects of inbreeding on the transgenerational expression of herbivore-induced defense traits in Solanum carolinense
Source: PLoS One. 2022 Oct 25;17(10):e0274920. doi: 10.1371/journal.pone.0274920 (PMC9595541; doi:10.1371/journal.pone.0274920)
Supplement: S3 Table — Compounds are in alphabetical order. Differences in quantities between maternal herbivory treatments and maternal breeding were determined by two-way ANOVAs and boldface indicate P < 0.1. (DOCX) [file pone.0274920.s003.docx]

**S3 Table.** **Focal compounds from induced volatile emissions of *S. carolinense* offspring.** Compounds are in alphabetical order. Differences in quantities between paternal damage treatments and maternal breed type were determined by two-way ANOVAs and boldface indicate *P* < 0.1.

|  | **Parental treatment** | | **Maternal breeding type** | |
| --- | --- | --- | --- | --- |
| **Compound** | **Damaged** | **Undamaged** | **Outbred** | **Inbred** |
| (-)-aristolene | 0.85 ± 0.23 | 2.40 ± 0.95 | 1.22 ± 0.39 | 2.14 ± 1.00 |
| α-cedrene | 1.05 ± 0.34 | 1.08 ± 0.34 | 1.24 ± 0.41 | 0.88 ± 0.24 |
| α-farnesene | 8.49 ± 2.40 | 7.08 ± 2.29 | 6.62 ± 1.96 | 8.85 ± 2.65 |
| α-guaiene | 0.57 ± 0.22 | 1.43 ± 0.77 | 0.67 ± 0.17 | 1.39 ± 0.85 |
| α-humulene | 2.55 ± 0.79 | 4.95 ± 2.28 | 4.20 ± 2.29 | 3.47 ± 1.23 |
| α-selinene | 0.94 ± 0.27 | 2.73 ± 1.13 | 1.28 ± 0.40 | 2.52 ± 1.20 |
| α-springene | 18.65 ± 7.20 | 18.75 ± 7.34 | 23.94 ± 8.34 | 13.47 ± 5.74 |
| α-terpinene | 0.95 ± 0.33 | 0.78 ± 0.28 | 1.14 ± 0.37 | 0.58 ± 0.18 |
| β-caryophyllene | **0.81 ± 0.29** | **0.35 ± 0.09** | 0.43 ± 0.14 | 0.70 ± 0.26 |
| β-elemene | 12.50 ± 3.25 | 30.55 ± 16.51 | **29.84 ± 16.74** | **14.49 ± 6.82** |
| β-ocimene | 27.91 ± 6.49 | 28.18 ± 6.80 | 27.23 ± 6.89 | 28.88 ± 6.46 |
| β-springene | 36.08 ± 13.98 | 40.28 ± 16.46 | 49.10 ± 18.32 | 27.57 ± 11.30 |
| 2-methyl-1-hepten-6-one | 0.96 ± 0.43 | 0.60 ± 0.25 | 0.42 ± 0.07 | 1.11 ± 0.46 |
| 4,11-selinadiene | 2.44 ± 0.76 | 5.38 ± 2.43 | 2.95 ± 0.79 | 5.09 ± 2.62 |
| butyl acetate | 1.55 ± 0.43 | 2.67 ± 1.03 | 2.36 ± 1.10 | 1.94 ± 0.45 |
| benzhydrazide | 0.86 ± 0.26 | 1.58 ± 0.90 | 1.76 ± 0.97 | 0.73 ± 0.14 |
| benzyl alcohol | 5.01 ± 1.15 | 3.04 ± 0.97 | 3.70 ± 1.22 | 4.21 ± 0.94 |
| caryophyllene oxide | 4.05 ± 1.33 | 3.36 ± 1.32 | 4.84 ± 1.69 | 2.52 ± 0.69 |
| longipinocarvone | 8.36 ± 2.24 | 15.55 ± 6.91 | 13.82 ± 6.81 | 10.60 ± 3.83 |
| compound 14 | 0.20 ± 0.05 | 0.19 ± 0.06 | 0.18 ± 0.06 | 0.20 ± 0.06 |
| compound 23 | 0.86 ± 0.28 | 0.90 ± 0.40 | 1.16 ± 0.44 | 0.61 ± 0.21 |
| compound 25 | 1.42 ± 0.38 | 3.54 ± 2.02 | **3.47 ± 2.05** | **1.64 ± 0.83** |
| decanal | **0.43 ± 0.29** | **0.22 ± 0.15** | 0.38 ± 0.27 | 0.26 ± 0.15 |
| epi-bicyclo-sesquiphellandrene | 1.52 ± 0.77 | 3.86 ± 1.74 | 2.24 ± 1.02 | 3.31 ± 1.77 |
| (*E*)-3-hexen-1-ol | 92.48 ± 31.76 | 129.90 ± 49.28 | 145.82 ± 56.32 | 79.23 ± 19.43 |
| (*E*)-4,8-Dimethyl-1,3,7-nonatriene | 287.45 ± 37.79 | 282.32 ± 40.42 | 317.11 ± 43.69 | 252.30 ± 32.24 |
| geraniolene | 395.39 ± 85.80 | 497.34 ± 138.43 | 533.63 ± 129.36 | 366.38 ± 104.94 |
| geranyl linallol | 53.84 ± 25.79 | 44.26 ± 24.10 | 62.66 ± 27.29 | 34.75 ± 21.65 |
| geranylacetone | 1.12 ± 0.56 | 0.76 ± 0.36 | 0.46 ± 0.08 | 1.39 ± 0.62 |
| methyl salicylate | 293.28 ± 95.93 | 372.69 ± 178.03 | 454.56 ± 193.29 | 217.08 ± 71.92 |
| m-ethylacetophenone | 0.27 ± 0.02 | 0.23 ± 0.03 | 0.26 ± 0.02 | 0.25 ± 0.03 |
| nerolidol | 31.36 ± 10.72 | 24.03 ± 7.10 | 32.08 ± 8.98 | 22.78 ± 8.64 |
| phenylethyl alcohol | 1.75 ± 0.45 | 1.67 ± 0.72 | 2.06 ± 0.84 | 1.36 ± 0.19 |
| sesquithujene | 1.27 ± 0.36 | 0.96 ± 0.35 | 1.29 ± 0.44 | 0.92 ± 0.23 |
| (Z)-3-hexen-1-ol | 6.00 ± 1.67 | 6.68 ± 3.64 | 9.06 ± 3.90 | 3.67 ± 1.17 |
| (*Z*)-3-hexen-1-ol, benzoate | 4.92 ± 1.11 | 5.29 ± 1.82 | 5.98 ± 2.01 | 4.26 ± 0.85 |
|  |  |  |  |  |
| **Average** | 1308.17 ± 272.15 | 1545.58 ± 428.61 | 1749.14 ± 471.98 | 1121.56 ± 199.25 |
