# Supplementary material for: Adverse effects of inbreeding on the transgenerational expression of herbivore-induced defense traits in Solanum carolinense
Source: PLoS One. 2022 Oct 25;17(10):e0274920. doi: 10.1371/journal.pone.0274920 (PMC9595541; doi:10.1371/journal.pone.0274920)
Supplement: S4 Table — Linear mixed-effects ANOVA for the effects of maternal herbivory (damage), maternal breeding, and their interaction on constitutive and induced levels of jasmonic acid (JA) and three JA-associated genes, allene oxide synthase (AOS), oxophytodienoate reductase-3 (OPR3), and lipoxygenase (LOX) in S. carolinense offspring. There were no significnat differenes. (DOCX) [file pone.0274920.s004.docx]

**S4 Table.** **Jasmonic acid and JA-associated genes.** Linear mixed-effects ANOVA for the effects of previous generation herbivore damage, maternal plant breeding type, and their interaction on constitutive and induced levels of jasmonic acid (JA) and three JA-associated genes, a*llene oxide synthase* (AOS), *oxophytodienoate reductase-3* (OPR3), and *lipoxygenase* (LOX) in *S. carolinense* offspring. There were no significnat differenes.

| *Target* | *Expression* | *Source of variation* | *Df* | *SS* | *F* | *P* |
| --- | --- | --- | --- | --- | --- | --- |
| **JA** | Constitutive | Damage | 1 | 0.001 | 0.632 | 0.433 |
|  |  | Breeding | 1 | 0.000 | 0.003 | 0.955 |
|  |  | Breeding x Damage | 1 | 0.000 | 0.061 | 0.807 |
|  |  | Error | 30 | 0.047 |  |  |
|  |  |  |  |  |  |  |
|  | Induced | Damage | 1 | 0.021 | 0.091 | 0.765 |
|  |  | Breeding | 1 | 0.067 | 0.297 | 0.586 |
|  |  | Breeding x Damage | 1 | 0.027 | 0.119 | 0.733 |
|  |  | Error | 30 | 6.737 |  |  |
| **AOS** | Constitutive | Damage | 1 | 0.001 | 0.010 | 0.921 |
|  |  | Breeding | 1 | 0.013 | 0.126 | 0.727 |
|  |  | Breeding x Damage | 1 | 0.018 | 0.180 | 0.676 |
|  |  | Error | 19 | 1.889 |  |  |
|  |  |  |  |  |  |  |
|  | Induced | Damage | 1 | 0.000 | 0.000 | 0.996 |
|  |  | Breeding | 1 | 0.113 | 1.303 | 0.268 |
|  |  | Breeding x Damage | 1 | 0.037 | 0.421 | 0.524 |
|  |  | Error | 19 | 1.654 |  |  |
| **LOX** | Constitutive | Damage | 1 | 0.012 | 0.210 | 0.652 |
|  |  | Breeding | 1 | 0.100 | 1.691 | 0.209 |
|  |  | Breeding x Damage | 1 | 0.07 | 0.124 | 0.729 |
|  |  | Error | 19 | 1.127 |  |  |
|  |  |  |  |  |  |  |
|  | Induced | Damage | 1 | 0.073 | 0.788 | 0.386 |
|  |  | Breeding | 1 | 0.151 | 1.635 | 0.217 |
|  |  | Breeding x Damage | 1 | 0.1322 | 1.431 | 0.246 |
|  |  | Error | 19 | 1.756 |  |  |
| **OPR3** | Constitutive | Damage | 1 | 0.045 | 0.405 | 0.532 |
|  |  | Breeding | 1 | 0.005 | 0.049 | 0.828 |
|  |  | Breeding x Damage | 1 | 0.069 | 0.631 | 0.437 |
|  |  | Error | 19 | 2.094 |  |  |
|  |  |  |  |  |  |  |
|  | Induced | Damage | 1 | 0.025 | 0.220 | 0.645 |
|  |  | Breeding | 1 | 0.200 | 0.177 | 0.679 |
|  |  | Breeding x Damage | 1 | 0.052 | 0.460 | 0.506 |
|  |  | Error | 19 | 2.149 |  |  |
