# Supplementary material for: Adverse effects of inbreeding on the transgenerational expression of herbivore-induced defense traits in Solanum carolinense
Source: PLoS One. 2022 Oct 25;17(10):e0274920. doi: 10.1371/journal.pone.0274920 (PMC9595541; doi:10.1371/journal.pone.0274920)
Supplement: S5 Table — Linear mixed-effects ANOVAs for the effects of maternal herbivory (damage), maternal breeding, and their interaction on larval mass gain and relative growth rate (RGR) of third and fourth instar M. sexta larvae feeding on leaves of S. carolinense offspring. There were no significant differences. (DOCX) [file pone.0274920.s005.docx]

**S5 Table.** **Larval mass gain and relative growth rate.** Linear mixed-effects ANOVAs for the effects of previous generation herbivore damage, maternal breeding type, and their interaction on larval mass gain and relative growth rate (RGR) of third and fourth instar *M. sexta* larvae feeding on leaves of *S. carolinense* offspring. *P* values <0.05 are in boldface.There were no significant differences.

| *Trait* | *M. sexta instar* | *Source of variation* | *Df* | *SS* | *F* | *P* |
| --- | --- | --- | --- | --- | --- | --- |
| Mass gain | Third instar | Damage | 1 | 0.00134 | 2.238 | 0.136 |
|  |  | Breeding | 1 | 0.00199 | 3.320 | 0.069 |
|  |  | Breeding x Damage | 1 | 0.00033 | 0.541 | 0.463 |
|  |  | Error | 285 | 0.17123 |  |  |
|  |  |  |  |  |  |  |
|  | Fourth instar | Damage | 1 | 0.00956 | 2.169 | 0.142 |
|  |  | Breeding | 1 | 0.00022 | 0.051 | 0.822 |
|  |  | Breeding x Damage | 1 | 0.00096 | 0.217 | 0.642 |
|  |  | Error | 309 | 1.36139 |  |  |
|  |  |  |  |  |  |  |
|  |  |  |  |  |  |  |
| RGR | Third instar | Damage | 1 | 0.06940 | 1.093 | 0.297 |
|  |  | Breeding | 1 | 0.06786 | 1.069 | 0.302 |
|  |  | Breeding x Damage | 1 | 0.04665 | 0.735 | 0.392 |
|  |  | Error | 285 | 18.0957 |  |  |
|  |  |  |  |  |  |  |
|  | Fourth instar | Damage | 1 | 0.13393 | 1.799 | 0.181 |
|  |  | Breeding | 1 | 0.03293 | 0.442 | 0.507 |
|  |  | Breeding x Damage | 1 | 0.00063 | 0.008 | 0.927 |
|  |  | Error | 309 | 23.0051 |  |  |
