# Supplementary material for: Adverse effects of inbreeding on the transgenerational expression of herbivore-induced defense traits in Solanum carolinense
Source: PLoS One. 2022 Oct 25;17(10):e0274920. doi: 10.1371/journal.pone.0274920 (PMC9595541; doi:10.1371/journal.pone.0274920)
Supplement: S6 Table — Log-likelihood ratio test of independence for the effects of maternal herbivory (damage), maternal breeding, their interaction, and maternal plant family on survivorship to adulthood of M. sexta feeding on S. carolinense offspring. There were no significant differences. (DOCX) [file pone.0274920.s006.docx]

**S6 Table.** **Manduca sexta survivorship.** Log-likelihood ratio test of independence for the effects of previous generation herbivory, maternal breeding type, their interaction, and maternal plant family on survivorship to adulthood of *M. sexta* feeding on *S. carolinense* offspring. There were no significant differences.

| *Trait* | *Source of variation* | *Df* | *G^2^* | *P* |
| --- | --- | --- | --- | --- |
| Survivorship to adulthood | Damage | 1 | 0.425 | 0.515 |
|  | Breeding | 1 | 0.425 | 0.515 |
|  | Breeding x Damage | 3 | 1.343 | 0.719 |
|  | Family | 2 | 2.900 | 0.235 |
|  |  |  |  |  |
