# Supplementary figures and images for: Adverse effects of inbreeding on the transgenerational expression of herbivore-induced defense traits in Solanum carolinense
Source: PLoS One. 2022 Oct 25;17(10):e0274920. doi: 10.1371/journal.pone.0274920 (PMC9595541; doi:10.1371/journal.pone.0274920)

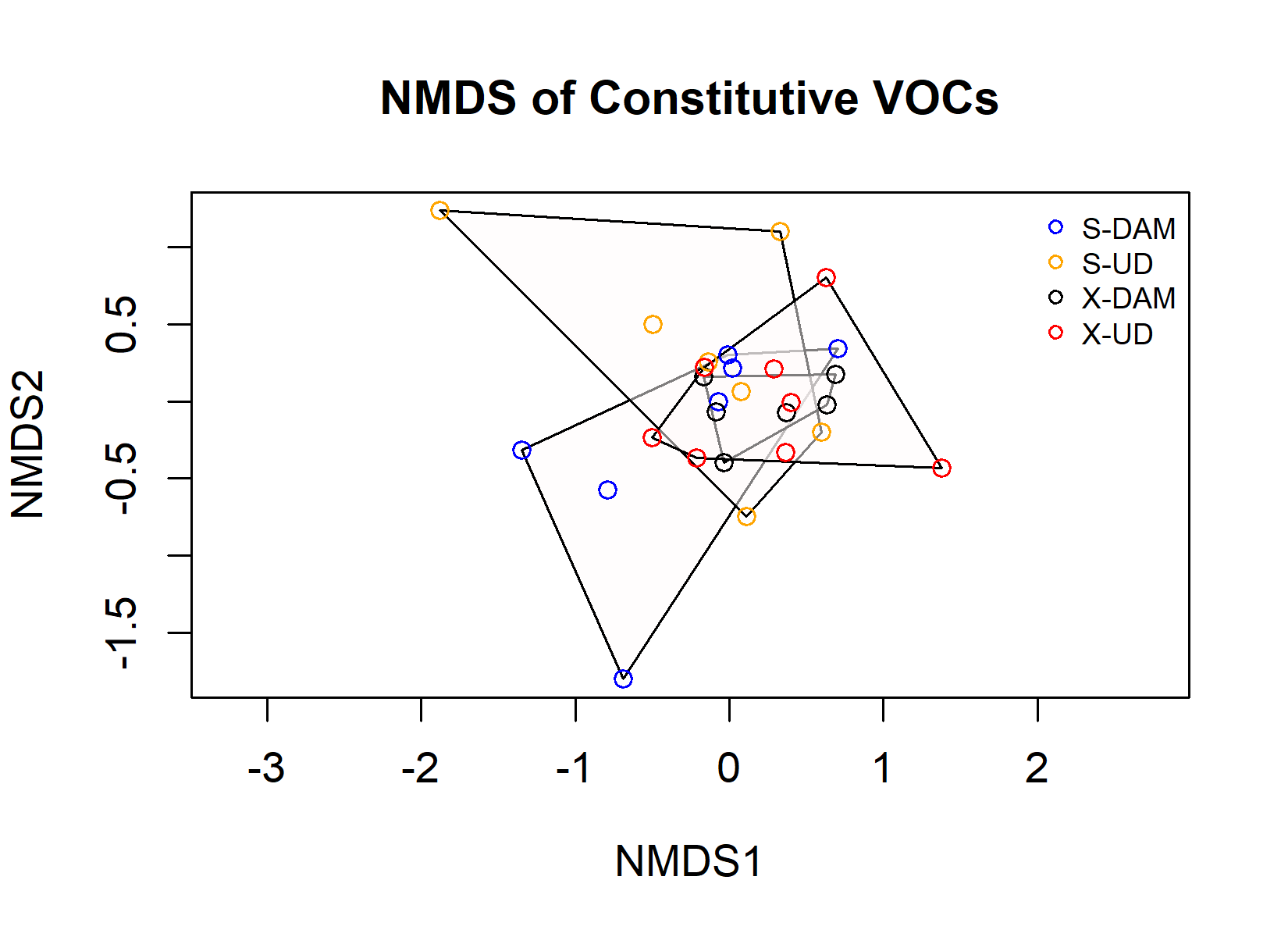

Supplement: S1 Fig — S-DAM = offspring of herbivore-damaged inbred maternal plants, S-UD = offspring of undamaged inbred maternal plants, X-DAM = offspring of herbivore-damaged outbred maternal plants, and X-UD = offspring of undamaged outbred maternal plants. (TIF) [file pone.0274920.s007.tif]

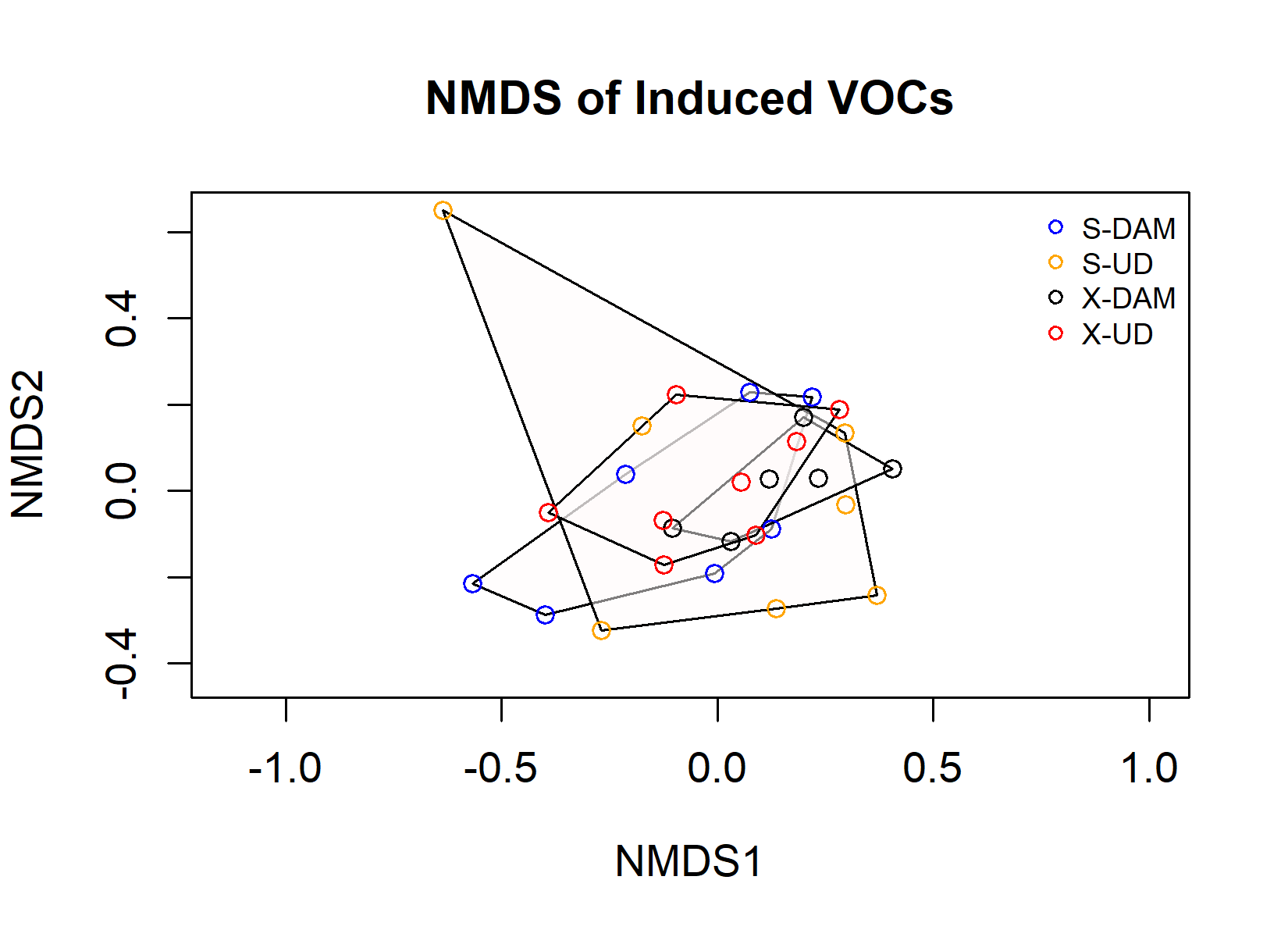

Supplement: S2 Fig — S-DAM = offspring of herbivore-damaged inbred maternal plants, S-UD = offspring of undamaged inbred maternal plants, X-DAM = offspring of herbivore-damaged outbred maternal plants, and X-UD = offspring of undamaged outbred maternal plants. (TIF) [file pone.0274920.s008.tif]

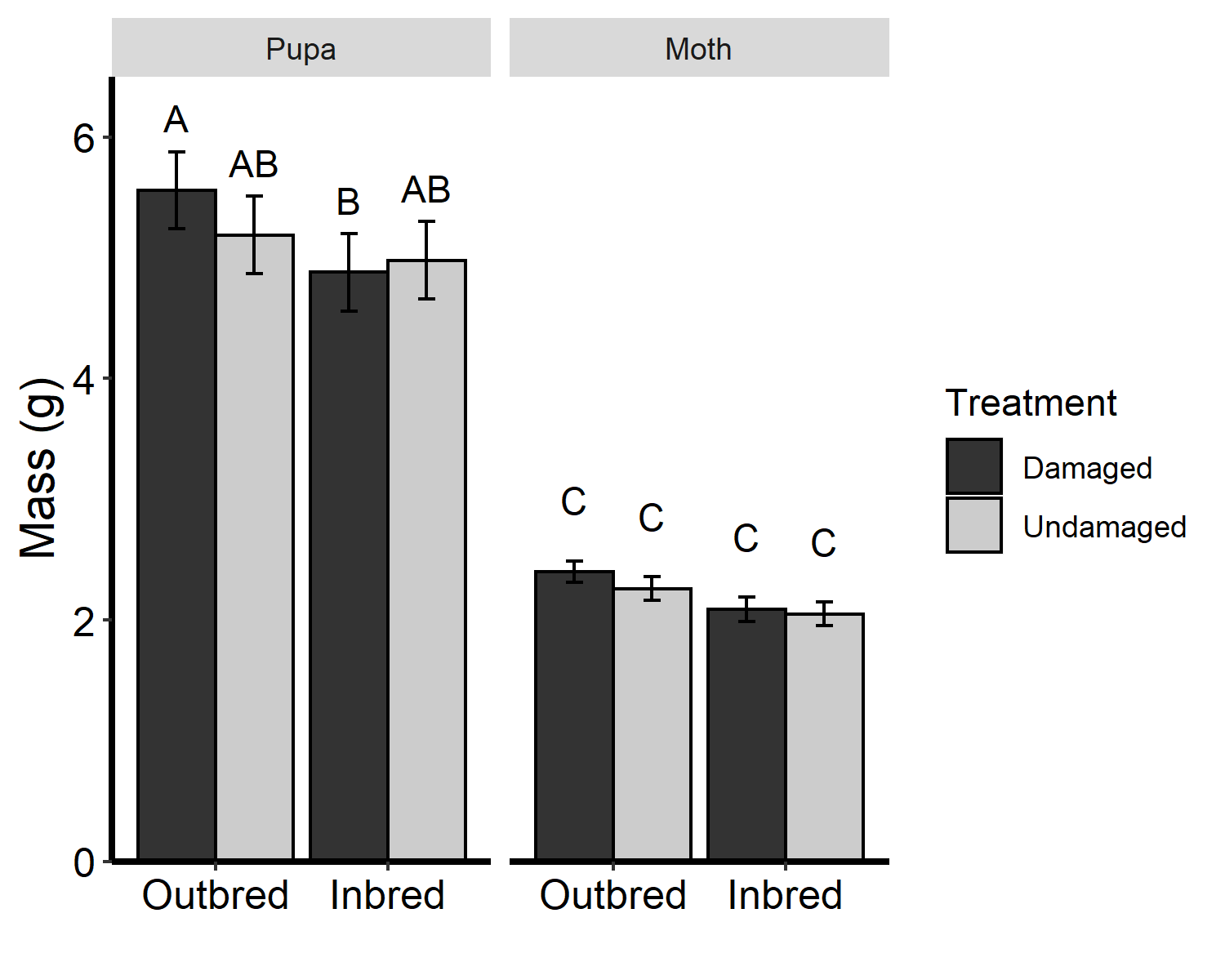

Supplement: S3 Fig — Different letters indicate significant differences among breeding by damage treatments determined by post hoc analysis using least square means multiple comparisons (P < 0.05). Error bars correspond to standard errors. (TIF) [file pone.0274920.s009.tif]

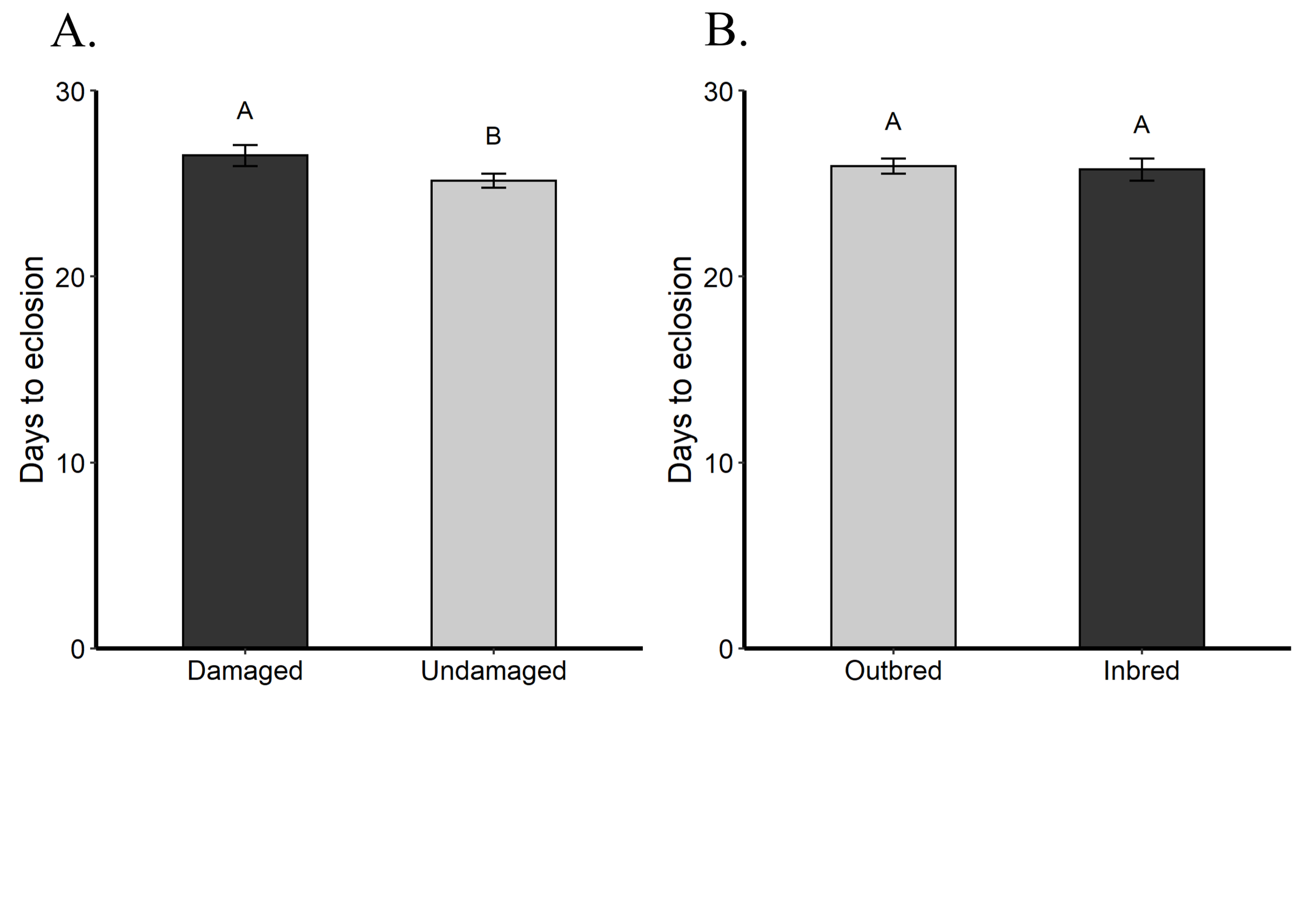

Supplement: S4 Fig — Different letters indicate significant differences among maternal breeding by maternal herbivory treatments determined by Wilcox signed-rank tests (P < 0.05). Error bars correspond to standard errors. (TIF) [file pone.0274920.s010.tif]
